# 《血栓性血小板减少性紫癜诊断与治疗中国指南（2022年版）》解读

**DOI:** 10.3760/cma.j.issn.0253-2727.2022.01.004

**Published:** 2022-01

**Authors:** 杰 殷, 自强 余

血栓性血小板减少性紫癜（thrombotic thrombocytopenic purpura, TTP）是由于血管性血友病因子裂解酶（ADAMTS13）活性缺乏引起广泛微血管血栓形成，导致微血管内溶血、消耗性血小板减少，心脑肾等脏器功能障碍的一种少见的血栓性微血管病。自2012年《血栓性血小板减少性紫癜诊断与治疗中国专家共识》公布以来，TTP的实验室检测、临床诊断、治疗方案的选择都有了明显的变化。因此中华医学会血液学分会血栓与止血学组经过讨论，发布了《血栓性血小板减少性紫癜诊断与治疗中国指南（2022版）》（简称指南）。在本文中，笔者针对指南中重要的更新部分作详细解读。

一、TTP的实验室检查

目前虽有多种ADAMTS13活性检测方法，但缺乏标准化[1]，且易受样本条件等因素影响，对可疑结果需重复送检。指南纳入了ADAMTS13-IgG抗体测定、基因检测、继发性TTP病因筛查和TTP患者重要器官功能的评估。其中，首次提出了采用ELISA法检测的ADAMTS13-IgG抗体测定。ADAMTS13的抗体分为两类，一类是抑制性抗体，与ADAMTS13结合后影响ADAMTS13的活性；另一类是非抑制性抗体，结合ADAMTS13后，加速ADAMTS13在血浆中的清除。ADAMTS13抗体绝大部分是IgG型，少部分是IgA和IgM型[2]。IgG抗体阳性对于ADAMTS13抑制物阴性的免疫性TTP（iTTP）患者具有重要诊断价值。ADAMTS13基因纯合突变或双重杂合突变可确诊遗传性TTP（cTTP）。对于ADAMTS13抑制物和ADAMTS13基因测序均阴性的患者，更倾向诊断iTTP。在诊断iTTP的同时，需要筛查感染、自身免疫性疾病等诱发因素[3]–[4]，故指南还纳入了乙型肝炎病毒（HBV）、丙型肝炎病毒（HCV）、人类免疫缺陷病毒（HIV）血清学检查，甲状腺功能，抗核抗体谱，狼疮抗凝物，抗磷脂抗体等检查项目。此外，TTP是一个多系统累及的疾病，因此需要对患者的心、脑、肾等重要脏器进行功能和影像学方面的系统检查，评估患者脏器的病变程度，还需要动态监测治疗后脏器功能的恢复情况。

二、TTP的临床诊断

ADAMTS13活性缺乏（<10％）是TTP最特异的诊断标准，但遗憾的是许多医院无法常规开展或者需要外送至其他检测中心完成，这不仅影响了患者诊断的及时性，也严重干扰了TTP患者的后续治疗。因此，指南推荐对于疑似TTP患者，可先行PLASMIC评分。我们先前的研究证实，PLASMIC积分在中国TTP患者中具有较高的诊断价值，积分为高度风险者诊断TTP的敏感性为87.5％，特异性为97.6％[5]。PLASMIC积分包括7项内容，这些检测结果在入院次日即可获得。对于积分达中高危患者应迅速开展血浆置换，及早改善病情。但是，需要注意的是PLASMIC积分的评估对象是血栓性微血管病（TMA）患者，主要是用于TMA的鉴别[6]。如果患者不符合TMA（微血管病性溶血、血小板减少和多脏器功能损害等），那么PLASMIC积分不适用。例如，Evans综合征患者PLASMIC积分通常也可达6分。

指南增加了TTP的诊断流程图。与其他疾病的诊断流程图不同的是，TTP患者的诊断与治疗是同时进行的。对于疑似患者，在送检ADAMTS13活性、抑制物或IgG抗体的同时，即开始血浆置换和糖皮质激素的治疗。根据ADAMTS13活性的结果，超过20％排除TTP，小于10％诊断TTP，10％～20％则需要根据病史来判断。对于诊断为TTP的患者，如ADAMTS13抑制物或IgG抗体阳性，确诊为iTTP，在原来治疗基础上加用利妥昔单抗和卡普赛珠单抗；反之则诊断为cTTP，可停用糖皮质激素和血浆置换，调整为定期血浆输注。iTTP一旦确诊，应尽早使用利妥昔单抗。这样的流程图更贴合TTP诊疗过程，有利于指导临床医生的实际工作。

三、TTP的治疗

1. 血浆置换：推荐使用新鲜（冰冻）血浆做血浆置换，不推荐冷沉淀上清或白蛋白配合晶体液。对于新诊断的TTP，冷沉淀上清做血浆置换增加TTP恶化风险[7]且可能引起获得性低纤维蛋白原血症，故需慎用。如每天1次血浆置换，患者症状仍进行性加重，可增加血浆置换的剂量或频次（如从每次2000 ml增加至3000 ml，或者每日行2次血浆置换）。患者临床症状缓解、血小板计数持续恢复正常2 d，可考虑终止血浆置换[8]–[9]。如血浆获取不受限，可先延长血浆置换间隔，然后逐步减停。在血浆置换联合利妥昔单抗治疗时，因血浆置换会损耗部分利妥昔单抗，因此尽可能拉长两者之间的间隔。推荐血浆置换后立即开始利妥昔单抗治疗，在利妥昔单抗结束至少20 h后再继续血浆置换[10]。

2. 卡普赛珠单抗：卡普赛珠单抗的主要作用是阻断血小板和VWF的结合，但是对于已形成的VWF-血小板复合物无效。因此，TTP患者越早使用，效果越明显。国外目前已开展采用卡普赛珠单抗联合重组ADAMTS13替代血浆置换治疗iTTP的临床试验，结果尚待揭晓。目前国内尚无此药，但同类产品即将开展临床试验。使用卡普赛珠单抗需注意的是，疗程要足够长，在停止血浆置换后仍需持续使用30 d[11]。需等到ADAMTS13活性完全恢复后停用，否则TTP病情会再次恶化[12]。

3. 硼替佐米：硼替佐米一则通过抑制浆细胞产生ADAMTS13抗体；二则通过抑制树突细胞的成熟减少ADAMTS13的内吞、减少抗原递呈、降低ADMATS13抗体[13]–[14]。硼替佐米对于复发、难治性TTP，尤其是对于利妥昔单抗治疗后复发的TTP患者，不失为一种很好的选择[15]。

4. 重组ADAMTS13：重组ADAMTS13在cTTP患者Ⅰ期和Ⅱ期临床试验中取得了不错的疗效[16]，Ⅲ期临床试验正在进行。用于治疗iTTP的临床试验也在进行中。国内也即将开展此类药物的临床试验。

5. 乙酰半胱氨酸（NAC）：NAC通过抑制VWF与VWF之间自联、VWF和血小板结合发挥作用，但对已形成的VWF多聚体和VWF-血小板复合物无效。因此NAC是TTP的辅助用药，推荐尽早使用改善TTP患者的临床症状。NAC需要持续大剂量使用，最佳使用方式是静脉给药，持续时间超过18 h[4]。但是，由于TTP患者还需要血浆置换和使用其他药物，故推荐尽可能长时间维持。

6. 治疗方案的调整：指南对于复发iTTP、缓解期cTTP及妊娠期TTP的治疗方案选择给出相应的推荐。对于cTTP患者，发病年龄越小，器官损伤越重，建议预防治疗。而cTTP孕妇则建议需血浆输注或血浆置换，积极行预防治疗[12]。cTTP孕妇妊娠期应该密切监测ADAMTS13活性和血小板变化，孕后期需提升血浆输注的剂量或调整为血浆置换以维持血小板数量在正常范围。iTTP缓解期患者如有妊娠需求，孕前应检查ADAMTS13活性，如ADMATS13活性低于10％，建议先行利妥昔单抗治疗，治疗结束至少6个月后再考虑妊娠[17]。妊娠期iTTP患者建议密切监测ADAMTS13活性，一旦低于10％即开始血浆置换。妊娠期iTTP患者不推荐利妥昔单抗治疗。

四、TTP的预后

指南的预后部分增加了一些新名词。除保留原有的临床缓解、临床复发等概念外，首次引入ADAMTS13缓解、ADAMTS13复发概念，强调了ADAMTS13测定在疗效评估和预后评估中的主要作用。也首次提出了难治性TTP的定义：去除诱因后，经5次血浆置换联合糖皮质激素治疗无临床反应（血小板计数持续低于50×10^9^/L，并且乳酸脱氢酶持续大于1.5倍正常值上限）[18]。提示这类患者体内抑制物滴度更高，且对糖皮质激素反应不佳，需要及早开始足量利妥昔单抗治疗。ADAMTS13复发是指iTTP患者尚无临床表现但ADAMTS13活性再次下降至20％以下，通常是临床复发的早期，应及早干预[19]。

由于TTP是一个多系统受累的疾病，发病时心、脑、肾等重要脏器易出现功能障碍。许多患者需要先在重症监护治疗病房行血浆置换等措施挽救生命，待病情稳定后再转入血液科进一步治疗。妊娠期TTP不仅涉及孕妇，也危及胎儿生命。因此，需要血液科、重症监护病房、神经科、妇产科、新生儿科、肾内科、心脏科等多学科团队密切合作，才能提供恰当的治疗方案，更好地挽救TTP患者的生命。
